# Central Serous Chorioretinopathy and Blood Serotonin Concentrations

**DOI:** 10.3390/jcm10040558

**Published:** 2021-02-03

**Authors:** Takeshi Kimura, Takashi Araki, Yuki Komuku, Hisashi Iwami, Fumi Gomi

**Affiliations:** Department of Ophthalmology, Hyogo College of Medicine, Nishinomiya 6638501, Japan; kimutake@hyo-med.ac.jp (T.K.); takas4i_cartar@hotmail.com (T.A.); yuki.kom0923@gmail.com (Y.K.); piwhiss@gmail.com (H.I.)

**Keywords:** central serous chorioretinopathy (CSC), serotonin, 5-hydroxytryptamine (5-HT), subfoveal choroidal thickness (SCT)

## Abstract

Background: To investigate blood serotonin (5-hydroxytryptamine (5-HT)) concentrations and their relationships with selected characteristics in patients with central serous chorioretinopathy (CSC). Methods: This was a prospective study including 93 patients with active CSC. Blood concentrations of 5-HT, adrenocorticotropic hormone, and cortisol were measured in patients with CSC. Selected patient characteristics, including disease history (acute or chronic), medication use, smoking history, mood status, best-corrected visual acuity (BCVA), subfoveal choroidal thickness (SCT), findings on fluorescein and indocyanine green angiography, and anatomical changes were evaluated during follow-up. Results: Eleven of the 93 patients had low 5-HT concentrations (<57 ng/mL) (12%, eight men and three women; mean age 55 years); we identified no significant relationship with acute/chronic disease status. The patients with low 5-HT were significantly more likely to have five or more fluorescein leakage sites (*p* = 0.0275), recurrence of subretinal fluids (*p* < 0.0001), and failure to achieve significant improvement in BCVA during follow-up (*p* = 0.862) than patients with 5-HT within the normal range. Conclusions: Blood serotonin concentrations may influence the pathophysiology and prognosis of CSC.

## 1. Introduction

Central serous chorioretinopathy (CSC), a common eye disease, is characterized by serous retinal detachment (SRD) in the macular area. Eyes with CSC show one or more leakages on fluorescein angiography (FA) and evidence of choroidal hyperpermeability on indocyanine green angiography (ICGA) [1,2]. Patients with CSC have thicker choroidal tissue than healthy individuals [3]. Psychological stress, Type-A behavior pattern, the use of steroids, and pregnancy are reportedly risk factors for CSC [4]. Although the pathophysiology of CSC is poorly understood, those risk factors suggest that the hypothalamic–pituitary–adrenal (HPA) axis plays an important role in this disease [5].

Previously, concentrations of steroid hormones, such as cortisol, aldosterone, and testosterone, have been widely investigated in patients with CSC; however, the findings have not been consistent, showing associations in some studies but not in others [6,7,8,9,10,11,12,13,14,15,16]. Serotonin (5-hydroxytryptamine (5-HT)), a neurotransmitter, influences many brain functions and plays important roles in the gastrointestinal system, blood, and brain, affecting behavior and physiology [17]. Whole blood serotonin concentrations are associated with a Type-A behavior pattern [18]. Additionally, a recent report by Sakai et al. suggested a possible relationship between lower blood 5-HT concentrations and chronic CSC [19]; however, this relationship has not been fully investigated. Hence, we hypothesized that 5-HT concentrations could affect the clinical characteristics in eyes with CSC, especially those with recurrences.

The purpose of this study was to investigate blood 5-HT concentrations and their relationships with selected characteristics in patients with CSC.

## 2. Materials and Methods

This prospective, observational case study was conducted at the Hyogo College of Medicine Hospital between June 2017 and November 2019. The Institutional Review Board of Hyogo College of Medicine approved this study (No. 3137), which complied with the guidelines of the Declaration of Helsinki.

Inclusion criteria included a diagnosis of active CSC, consent to testing of blood samples and responding to a questionnaire concerning lifestyle and mood, follow-up for more than 3 months from baseline, and the ability to provide written informed consent. Active CSC was diagnosed on the basis of detection of SRD on optical coherence tomography (OCT) and of idiopathic leaks from the retinal pigment epithelium during fluorescein angiography (FA). Indocyanine green angiography (ICGA) was performed to check for areas showing choroidal vascular hyperpermeability and to exclude age-related macular degeneration, polypoidal choroidal vasculopathy, and Vogt–Koyanagi–Harada disease. Exclusion criteria comprised: (1) other retinal or choroidal disease causing submacular fluid; (2) choroidal neovascularization (CNV) detected on OCT angiography (OCTA); (3) current treatment with systemic steroids; and (4) pregnancy or other systemic diseases affecting blood hormone concentrations.

All participants underwent complete ophthalmic examinations, including measurement of best-corrected visual acuity (BCVA), slit-lamp biomicroscopy, color fundus photography, fundus autofluorescence (FAF), FA and ICGA using a confocal scanning laser ophthalmoscope (HRA; Heidelberg Engineering, Heidelberg, Germany), and OCT and OCTA using a swept source OCT (SS-OCT) (DRI-OCT; Topcon, Tokyo, Japan). Visual acuity for each patient was examined using Landolt C chart under full correction of the refractive error with a distance of 5 m. The SCT was measured, being defined as the distance between the outer portion of the hyper-reflective line matching with the retinal pigment epithelium to the inner surface of the sclera on B-scan SS-OCT images. CSC was classified acute or chronic on the basis of an interview. Acute CSC was defined as of recent onset (visual symptoms within the previous 6 months), whereas chronic CSC was defined as chronic disease (symptoms for more than 6 months or having had one or more prior episodes of CSC).

Blood samples were collected to measure the concentrations of 5-HT, as well as cortisol, aldosterone, and ACTH around 6 h after awakening. The concentrations were determined at SRL (Tokyo, Japan) by high-performance liquid chromatography coupled with electrochemical detection. The reference ranges for cortisol, aldosterone, ACTH, and 5-HT are 6.24–18 µg/dL, 35.7–240 pg/mL, 7.2–63.3 pg/mL, and 57–230 ng/mL, respectively.

The CSC patients’ moods were assessed by responses to a questionnaire, the Japanese version of the Profile of Mood States 2nd Edition (POMS2) [20]. The questionnaire provides scores for anger/hostility (AH), confusion/bewilderment (CB), depression/dejection (DD), fatigue/inertia (FI), tension/anxiety (TA), vigor/activity (VA), friendliness (F), and total mood disturbance (TMD). The TMD score, a comprehensive evaluation of negative mood state, is calculated as follows: TMD = {[AH] + [CB] + [DD] + [FI] + [TA]} − [VA]. The mean TMD score for normal volunteers is approximately 50; a higher the TMD score, the more negative the mood state.

A structured interview ascertaining history of medications and smoking, sleep patterns, and occupational status was also conducted. Sleep disturbance was defined with or without sleep medications. The definition of smoking included past and current smokers.

All examinations were performed at baseline. When SRF had persisted more than 3 months after the current onset, laser photocoagulation or reduced-fluence photodynamic therapy (rfPDT) was administered according to the site(s) of fluorescein leakage. Reduced-fluence PDT for CSC was approved by the institutional review board of our hospital. All patients were followed for more than 3 months from baseline and BCVA was assessed and OCT performed at every visit. Recurrence was defined as reappearance of submacular fluid on OCT after treatment-related or spontaneous resolution.

In cases of bilateral involvement, data from the eye with more subretinal fluid at the macula were included in the analysis. JMP^®^ pro 14 (SAS Institute, Cary, NC, USA) was used for statistical analysis. The decimal BCVA was converted to the logarithm of minimum angle of resolution (logMAR) units for statistical analysis. Numerical variables are expressed as the mean and standard deviation. Categorical variables were analyzed using the χ^2^ test. The Wilcoxon’s rank test was used to compare variables between the two groups. Paired *t*-tests were used to compare variables at baseline and after treatment. We used logistic regression analysis to examine the factors that affect 5-HT concentrations among the participants, and multivariate analysis to explore the factors that influence the SCT and 5-HT concentrations. A *p* value of <0.05 was considered to denote statistical significance.

## 3. Results

**Figure 1:**
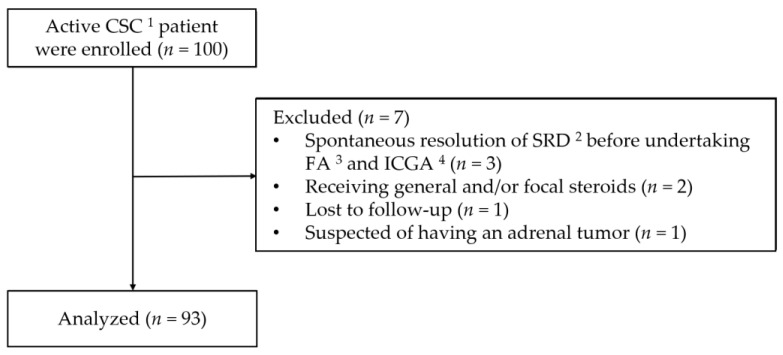
This study flow diagram. ^1^ central serous chorioretinopathy; ^2^ serous retinal detachment; ^3^ fluorescein angiography; ^4^ indocyanine green angiography.

Informed consent to participate in the present study was obtained from 100 patients. Seven of these patients were subsequently excluded for the following reasons: three had spontaneous resolution of SRD before undertaking FA and ICGA, two were later found to have been receiving general and/or focal steroids, one failed to attend for follow-up, and one was suspected of having an adrenal tumor on the basis of blood hormone concentrations. The study flow diagram is shown in Figure 1. Thus, the study cohort comprised 93 patients (74 men and 19 women; mean age, 50.1 years; age range, 31–75 years), 19 of whom had bilateral active CSC. Thirty-two patients were diagnosed as having acute and 61 chronic CSC.

The mean blood concentrations of aldosterone, cortisol, ACTH, and 5-HT were 155.0 ± 65.9 pg/mL, 9.4 ± 3.3 µg/dL, 24.1 ± 9.9 pg/mL, and 94.5 ± 38.5 ng/mL respectively and those values were within normal range. Three patients had aldosterone concentration above the higher limit of the normal range, 2 patients had cortisol concentration above its higher limit, no patients had higher ACTH and 11 patients (11.8%) had 5-HT concentrations below the lower limit.

Focusing on 5-HT, Table 1 showed the characteristics of patients in the low and normal 5-HT concentrations. The patients with low 5-HT concentrations tended to be older (*p* = 0.0574); three of them (27.3%) were taking psychiatric pharmacotherapy, which comprised selective serotonin reuptake inhibitors (SSRIs) in two of these three patients. Only two patients (2.4%) in the normal 5-HT concentration group were taking psychiatric pharmacotherapy other than SSRIs. The mean TMD score in this study was 45.4 points, which constituted a normal value. However, the mean TMD score was significantly higher in patients with acute CSC (56.5 points) than in patients with chronic CSC (39.7 points).

There were no significant differences in the concentration of aldosterone, cortisol and ACTH between low and normal 5-HT groups (*p* = 0.3234, *p* = 0.4717, *p* = 0.8351, respectively); however, significantly more eyes had five or more leakage points on FA (*p* = 0.0275) in the low 5-HT group.

The univariate logistic regression showed that patients receiving psychiatric pharmacotherapy, those with five or more leakage points on FA, and those with more subfoveal choroidal thickness were more likely to have 5-HT concentrations below the lower limit of normal range (Table 2).

**Figure 2:**
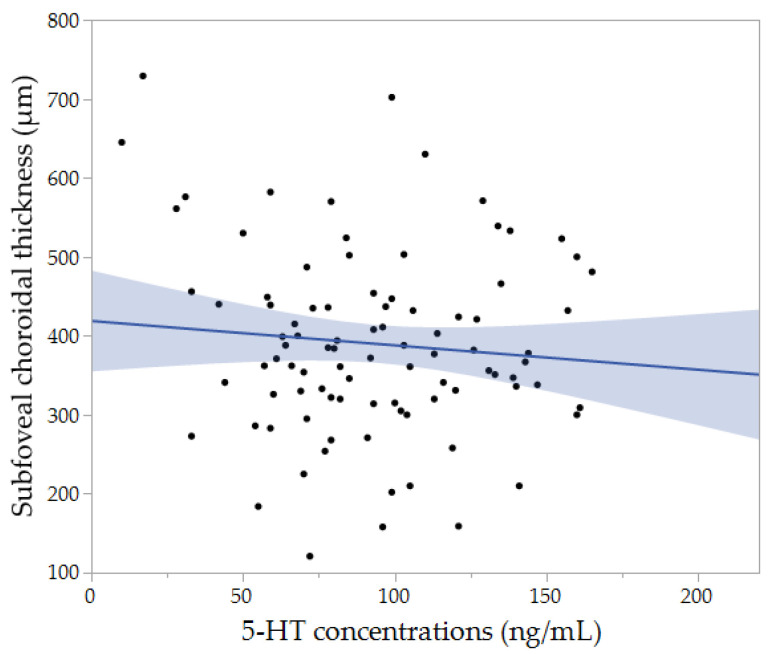
The association between subfoveal choroidal thickness and 5-HT concentrations; No association was found between subfoveal choroidal thickness and 5-HT concentrations (*r* = −0.056, *p* = 0.5937).

The multivariate analysis revealed that the SCT was significantly associated with younger age (*p* = 0.0105) and more spherical equivalent (*p* = 0.0071). After adjusting for age and spherical equivalent, the multiple regression analysis showed that the patients with 5-HT concentrations below the lower limit of normal were more likely to have higher SCT (*p* = 0.0121). Then, we performed the correlation analysis to know any relationships between 5-HT concentrations and SCT, but it did not show the significant association (*r* = −0.056, *p* = 0.5937) (Figure 2).

The mean duration of follow-up was 9.9 ± 8.4 months (range: 3–34 months). Laser photocoagulation was administered to 26 eyes and rfPDT to 58 eyes and 91 of 93 eyes were found to have dry macula on at least one visit. Table 3 shows the BCVA at baseline and final examination and the changes during follow-up in eyes with normal and low 5-HT concentrations. Baseline and final BCVA values, as well as changes in BCVA during follow-up, did not significantly differ on the basis of 5-HT concentration. Patients with normal 5-HT concentrations exhibited improvement in final BCVA, compared with baseline (*p* < 0.0001), while final BCVA did not differ significantly from baseline values in patients with low 5-HT concentrations (*p* = 0.861).

**Figure 3:**
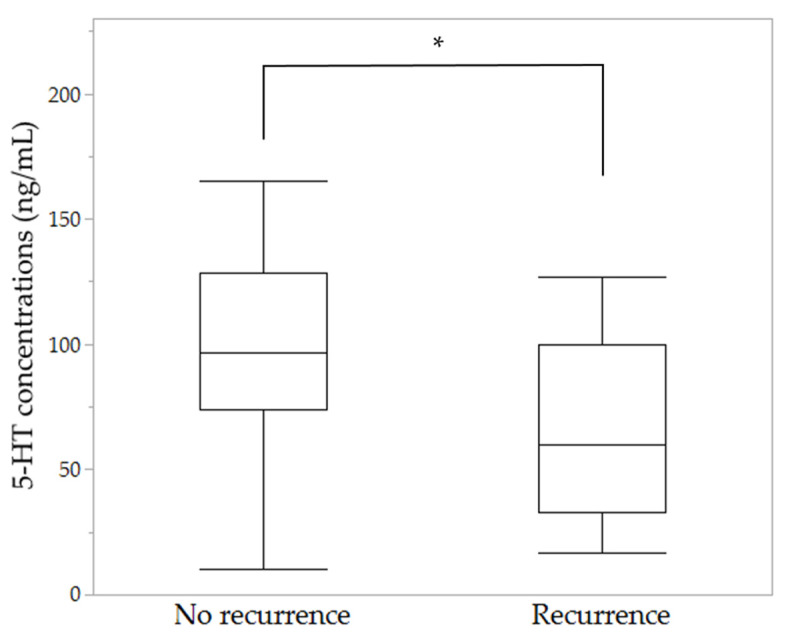
Comparison of 5-HT concentrations based on the presence of CSC recurrences; 5-HT concentrations were significantly lower in patients with CSC recurrence (* *p* = 0.001).

Recurrences were seen in 17 cases: one (3.1%) with acute CSC and 16 (26.2%) with chronic CSC; this difference was statistically significant (*p* = 0.0062). In addition, 10 patients (12.2%) with normal 5-HT concentrations and seven (63.6%) with low 5-HT concentrations had one or more recurrences, the recurrence rate in those with low 5-HT concentrations being significantly higher than in those with normal 5-HT concentrations (*p* < 0.0001). The mean 5-HT concentrations were 100.9 ± 36.6 ng/mL in the patients without recurrences and 65.9 ± 34.0 ng/mL in those with recurrence; the 5-HT concentrations in the patients with recurrence were significantly lower than those without recurrences. (*p* = 0.001) (Figure 3).

## 4. Discussion

CSC is known to be strongly associated with exogenous and/or endogenous high glucocorticoids, Type-A behavior, and psychological distress [4,21,22]. It has been reported that exogenous steroids can cause more severe phenotypes of CSC, manifesting as bilateral involvement, a higher frequency of pigment epithelial detachment, and multiple leakage sites on FA with thicker choroid [23]. Psychological distress may be associated with increased endogenous activity of the HPA axis [5,21,24,25]; however, the relationship between the HPA system and the pathogenesis of CSC is unclear. We conducted this study to investigate blood 5-HT concentrations and their relationships with selected characteristics in patients with CSC, for the purpose of being to enable earlier identification of individuals at high risk of developing this disease.

Our findings suggest that 5-HT plays a significant role in determining the characteristics of CSC. For example, reduced 5-HT concentrations have been observed in patients with depression and panic attacks [26]. With respect to CSC, Sakai et al. reported a mean blood 5-HT concentration of 98.2 ± 27.5 ng/mL in 34 patients with chronic CSC, which was significantly lower than those in 31 normal controls (128.5 ± 35.8 ng/mL) and 15 patients with acute CSC (122.5 ± 23.9 ng/mL) [19]. In our study, the mean 5-HT concentration in 93 patients with CSC was 94.5 ± 38.5 ng/mL, which is lower than that in the normal controls in Sakai et al.’s study. In our study, there was no significant difference in 5-HT concentrations between the acute and chronic group. This discrepancy may be attributable to differences in the definitions of acute and chronic CSC; Sakai et al. defined self-resolving SRD within 6 months from the onset of symptoms as acute CSC, whereas we defined patients with symptoms within the 6 months prior to the blood test as having acute CSC, some of them would have been defined as chronic in Sakai et al.’s study.

In a previous study, three of 49 CSC patients (6.1%) had 5-HT concentrations below the normal range [19], whereas our study included a greater proportion of patients with low 5-HT concentrations: 11 of 93 (11.8%). Serotonin is a neurotransmitter that works in the brain and modifies the effects of sympathetic activity. Low blood 5-HT concentrations may lead to an imbalance between the sympathetic and parasympathetic nervous systems. Previous studies have shown significantly lower blood 5-HT concentrations in shift workers than day workers [27]; this hormone plays an important role in sleep pathophysiology [28]. In addition, psychiatric disorders are reportedly associated with reduced 5-HT activity [29]. For example, patients with depression reportedly have lower 5-HT concentrations than normal volunteers [30]; the mean 5-HT concentration for such patients is reportedly 73.75 ng/mL [31]. Conversely, a previous study reported administration of SSRIs lead to elevated 5-HT concentrations [32]. Although the use of SSRIs may thus lead to elevated 5-HT concentrations, two patients in the low 5-HT group were taking SSRIs in our study. Thus, we presumed that the use of SSRIs did not affect our results. In addition, one patient in the low 5-HT group was taking a noradrenergic and specific serotonergic antidepressant in our study. To the best of our knowledge, there has been no report concerning the relationship between 5-HT concentrations and the use of noradrenergic and specific serotonergic antidepressants; thus, we cannot rule out the possibility that the use of such medication may have affected the 5-HT concentration in our patient. Thus, such medications may be directly or indirectly associated with development of CSC.

In this study, patients with low 5-HT had higher frequency of having five or more leakage sites on FA and the more SCT than those with normal 5-HT. Although we found no association between acute/chronic disease status and normal/low 5-HT concentrations, the cases with low 5-HT concentrations had a significantly higher rate of recurrence and failed to achieve significant improvement in vision. Several factors such as greater subfoveal choroidal thickness, nonintense fluorescein leakage, shift work, and a history of psychiatric illness such as depression, sleep disorders, and adjustment disorder are known risk factors for CSC recurrence [33,34,35]. We have previously reported that patients with steroid-related CSC have more fluorescein leakage sites (*p* < 0.0001), greater choroidal thickness (*p* = 0.0287) and a higher recurrence rate (*p* = 0.0412) than those with non-steroid related CSC [23] and these characteristics are similar to those of individuals with low 5-HT concentrations. As has long been suggested, a common underlying mechanism may link abnormalities in the 5-HT system and HPA axis hyper-reactivity to psychological stress [36]. Low blood 5-HT concentrations may reflect general systemic vascular dysregulation, as observed in patients with hypertension [37]. In the presence of significant imbalance between the sympathetic and parasympathetic nervous systems, the choroidal vessels fail to maintain homeostasis [38], resulting in the choroidal vascular hyperperfusion and congestion that is frequently seen in CSC. Our results support the contention that low blood 5-HT concentrations may influence choroidal circulation in CSC patients. However, a previous study showed associations between SCT and the levels of some cytokines in patients with CSC [39], thus, more research is needed to more precisely identify the underlying mechanisms.

Treatment may result in visual improvement in eyes with CSC [4]. However, Fok et al. reported that untreated patients with CSC with one or more episodes of recurrence do not achieve a statistically significant improvement over baseline values [36]. Therefore, recurrences can be associated with failure to achieve significant improvement in BCVA in CSC patients and low 5-HT concentrations may be one of the risk factors. We propose that the measurement of blood 5-HT concentrations in patients with CSC may aid in determining the future recurrence risk and vision prognosis.

The limitations of this study include the small sample and resultant small number of patients with low 5-HT concentrations. Furthermore, it lacked a control group and used definitions of acute and chronic CSC that differed from those of an important prior study [19]. Notably, there have been no consistent definitions of chronic and acute CSC. Therefore, in this study, we stratified patients with CSC on the basis of their subjective symptom duration. As noted above, the sample size may have been inadequate. The previous study by Sakai et al. [19] compared 5-HT concentrations between 49 patients with CSC (15 with acute CSC and 34 with chronic CSC) and 30 age-matched controls. Because our study was not designed to compare 5-HT concentrations, we could not fully control the sample size. It may be possible to overcome these limitations by conducting multi-institutional joint research.

In conclusion, in this study we found that patients with CSC and low 5-HT concentrations had thicker choroid, higher recurrence rates, and no improvement in BCVA compared with those with normal 5-HT concentrations. Our findings may enable identification of new options for diagnosis/treatment of CSC on the basis of blood 5-HT concentrations.

## Figures and Tables

**Table 1:** Characteristics in the low and normal 5-HT concentrations.

	Low 5-HT ^1^ (*n* = 11)	Normal 5-HT ^1^ (*n* = 82)	*p*-Value
Age (years)	55.1 ± 10.2	49.4 ± 9.3	0.0574
Sex (male/female)	8/3	66/16	0.5489
Bilateral involvement (%)	3 (27.3)	16 (19.5)	0.5489
Acute/chronic	2/9	30/52	0.2276
Hypertension (*n*) (%)	4 (36.4)	24 (29.3)	0.6300
Sleep disturbance (*n*) (%)	1 (9.1)	6 (7.3)	0.8341
Psychiatric pharmacotherapy (*n*) (%)	3 (27.3)	2 (2.4)	0.0006 *
Smoking (*n*) (%)	8 (72.7)	55 (67.1)	0.7064
TMD ^2^ scores	37.2 ± 29.3	46.5 ± 34.0	0.4358
Spherical equivalent (diopters)			
Mean	−0.7 ± 2.6	−1.0 ± 2.0	0.6682
Fluorescein angiography findings			
One leakage point (*n*) (%)	3 (27.3)	43 (52.4)	0.1170
Two to four leakage points (*n*) (%)	2 (18.2)	20 (24.4)	0.6491
Five or more leakage points (*n*) (%)	6 (54.5)	19 (23.2)	0.0275 *
Indocyanine green angiography findings			
Choroidal vascular hyperpermeability (*n*) (%)	11 (100)	70 (85.4)	0.1740
Central retinal thickness (μm)			
Mean	285.5 ± 92.1	331.5 ± 114.8	0.2182
Subfoveal choroidal thickness (μm)			
Mean	456.5 ± 170.6	380.7 ± 105.4	0.1263

^1^ 5-hydroxytryptamine; ^2^ total mood disturbance; * *p* < 0.05.

**Table 2:** Factors associated with low 5-HT concentrations.

Variable	Odds Ratio	95% Confidence Interval	*p*-Value
Age (years)	1.059	0.995–1.126	0.0693
Psychiatric pharmacotherapy	15.0	2.175–103.464	0.006 *
Five or more fluorescein leakage points/one leakage point	4.526	1.023–20.031	0.0466 *
Subfoveal choroidal thickness (μm)	1.005	1.000–1.011	0.0458 *

* *p* < 0.05.

**Table 3:** Changes in best corrected visual acuity.

	Low 5-HT ^1^	Normal 5-HT ^1^	*p*-Value
BCVA ^2^ at baseline	0.153	0.0797	0.4240
BCVA ^2^ at final visit	0.144	−0.00445	0.1459
Changes in BCVA ^2^ between baseline and final visit	−0.00863	−0.0842	0.2613
*p* value	0.8616	<0.0001 *	

^1^ 5-hydroxytryptamine; ^2^ best-corrected visual acuity; * *p* < 0.05.

## Data Availability

The datasets analyzed during the current study are available from the corresponding author on reasonable request.

## References

[1] Guyer D.R., Yannuzzi L.A., Slakter J.S., Sorenson J.A., Ho A., Orlock D. (1994). Digital indocyanine green videoangiography of central serous chorioretinopathy. Arch. Ophthalmol..

[2] Gass J.D. (1967). Pathogenesis of disciform detachment of the neuroepithelium. Am. J. Ophthalmol..

[3] Kuroda S., Ikuno Y., Yasuno Y., Nakai K., Usui S., Sawa M., Tsujikawa M., Gomi F., Nishida K. (2013). Choroidal thickness in central serous chorioretinopathy. Retina.

[4] Daruich A., Matet A., Dirani A., Bousquet E., Zhao M., Farman N., Jaisser F., Behar-Cohen F. (2015). Central serous chorioretinopathy: Recent findings and new physiopathology hypothesis. Prog. Retin. Eye Res..

[5] Juruena M.F., Agustini B., Cleare A.J., Young A.H. (2017). A translational approach to clinical practice via stress-responsive glucocorticoid receptor signaling. Stem Cell Investig..

[6] Çiloğlu E., Unal F., Dogan N.C. (2018). The relationship between the central serous chorioretinopathy, choroidal thickness, and serum hormone levels. Graefe’s Arch. Clin. Exp. Ophthalmol..

[7] Tufan H.A., Gencer B., Comez A.T. (2013). Serum cortisol and testosterone levels in chronic central serous chorioretinopathy. Graefe’s Arch. Clin. Exp. Ophthalmol..

[8] Lotery A., Sivaprasad S., O’Connell A., Harris R.A., Culliford L., Ellis L., Cree A., Madhusudhan S., Behar-Cohen F., Chakravarthy U. (2020). Eplerenone for chronic central serous chorioretinopathy in patients with active, previously untreated disease for more than 4 months (VICI): A randomised, double-blind, placebo-controlled trial. Lancet.

[9] Gong Q., Sun X., Yuan S., Liu Q. (2017). The relation of the serum aldosterone level and central serous chorioretinopathy—A pilot study. Eur. Rev. Med. Pharmacol. Sci..

[10] Schellevis R.L., Altay L., Kalisingh A., Mulders T.W.F., Sitnilska V., Hoyng C.B., Boon C.J.F., Groenewoud J.M.M., de Jong E.K., den Hollander A.I. (2019). Elevated Steroid Hormone Levels in Active Chronic Central Serous Chorioretinopathy. Investig. Ophthalmol. Vis. Sci..

[11] Agarwal A., Garg M., Dixit N., Godara R. (2016). Evaluation and correlation of stress scores with blood pressure, endogenous cortisol levels, and homocysteine levels in patients with central serous chorioretinopathy and comparison with age-matched controls. Indian J. Ophthalmol..

[12] Natung T., Keditsu A. (2015). Comparison of Serum Cortisol and Testosterone Levels in Acute and Chronic Central Serous Chorioretinopathy. Korean J. Ophthalmol..

[13] Garg S.P., Dada T., Talwar D., Biswas N.R. (1997). Endogenous cortisol profile in patients with central serous chorioretinopathy. Br. J. Ophthalmol..

[14] Zakir S.M., Shukla M., Simi Z.U.R., Ahmad J., Sajid M. (2009). Serum cortisol and testosterone levels in idiopathic central serous chorioretinopathy. Indian J. Ophthalmol..

[15] Chalisgaonkar C., Chouhan S., Lakhtakia S., Choudhary P., Dwivedi P.C., Rathore M.K. (2010). Central serous chorioretinopathy and endogenous cortisol-is there an association. Indian J. Ophthalmol..

[16] van Haalen F.M., van Dijk E.H.C., Dekkers O.M., Bizino M.B., Dijkman G., Biermasz N.R., Boon C.J.F., Pereira A.M. (2018). Cushing’s syndrome and hypothalamic-pituitary-adrenal axis hyperactivity in chronic central serous chorioretinopathy. Front. Endocrinol. (Lausanne)..

[17] Bunney P.E., Zink A.N., Holm A.A., Billington C.J., Kotz C.M. (2017). Stress exposure and psychopathology alter methylation of the serotonin receptor 2A (HTR2A) gene in preschoolers. Dev. Psychopathol..

[18] Madsen D., McGuire M.T. (1984). Rapid communication whole blood serotonin and the Type A behavior pattern. Psychosom. Med..

[19] Sakai T., Tsuneoka H. (2017). Reduced Blood Serotonin Levels in Chronic Central Serous Chorioretinopathy. Ophthalmol. Retin..

[20] Heuchert J.P., McNair D.M., Yokoyama K., Watanabe K. (2015). Profile of Mood States.

[21] Scarinci F., Ghiciuc C.M., Patacchioli F.R., Palmery M., Parravano M. (2019). Investigating the Hypothesis of Stress System Dysregulation as a Risk Factor for Central Serous Chorioretinopathy: A Literature Mini-Review. Curr. Eye Res..

[22] Wang E., Chen S., Yang H., Yang J., Li Y., Chen Y. (2019). CHOROIDAL THICKENING and PACHYCHOROID in CUSHING SYNDROME: Correlation with Endogenous Cortisol Level. Retina.

[23] Araki T., Id H.I., Iwahashi C., Niki M., Mitamura Y., Sugimoto M., Kondo M., Kinoshita T. (2019). Central serous chorioretinopathy with and without steroids: A multicenter survey. PLoS ONE.

[24] Kumar M., Van Dijk E.H.C., Raman R., Mehta P., Boon C.J.F., Goud A., Bharani S., Chhablani J. (2020). Stress and vision-related quality of life in acute and chronic central serous chorioretinopathy. BMC Ophthalmol..

[25] Lahousen T., Painold A., Luxenberger W., Schienle A., Kapfhammer H.P., Ille R. (2016). Psychological factors associated with acute and chronic central serous chorioretinopathy. Nord. J. Psychiatry.

[26] Szeitz A., Bandiera S.M. (2018). Analysis and measurement of serotonin. Biomed Chromatogr..

[27] Sookoian S., Gemma C., Gianotti T.F., Burgueño A., Alvarez A., González C.D., Pirola C.J. (2007). Serotonin and serotonin transporter gene variant in rotating shift workers. Sleep.

[28] Ursin R. (2002). Serotonin and sleep. Sleep Med. Rev..

[29] Manchia M., Carpiniello B., Valtorta F., Comai S. (2017). Serotonin Dysfunction, Aggressive Behavior, and Mental Illness: Exploring the Link Using a Dimensional Approach. ACS Chem. Neurosci..

[30] Mahar I., Bambico F.R., Mechawar N., Nobrega J.N. (2014). Stress, serotonin, and hippocampal neurogenesis in relation to depression and antidepressant effects. Neurosci. Biobehav. Rev..

[31] Saldanha D., Kumar N., Ryali V., Srivastava K., Pawar A.A. (2009). Serum Serotonin Abnormality in Depression. Med. J. Armed. Forces India..

[32] De Groote L., Olivier B., Westenberg H.G. (2002). The effects of selective serotonin reuptake inhibitors on extracellular 5-HT levels in the hippocampus of 5-HT(1B) receptor knockout mice. Eur. J. Pharmacol..

[33] Matet A., Daruich A., Behar-cohen F. (2018). Risk factors for recurrences of central serous chorioretinopathy. Retina.

[34] Fok A.C.T., Chan P.P.M., Lam D.S.C., Lai T.Y.Y. (2011). Risk Factors for Recurrence of Serous Macular Detachment in Untreated Patients with Central Serous Chorioretinopathy. Ophthalmic. Res..

[35] Yu J., Xu G., Chang Q., Ye X., Li L., Jiang C., Zhao Q. (2019). Risk Factors for Persistent or Recurrent Central Serous Chorioretinopathy. J. Ophthalmol..

[36] Lanfumey L., Mongeau R., Cohen-Salmon C., Hamon M. (2008). Corticosteroid-serotonin interactions in the neurobiological mechanisms of stress-related disorders. Neurosci. Biobehav. Rev..

[37] Watts S.W., Morrison S.F., Davis R.P., Barman S.M. (2012). Serotonin and Blood Pressure Regulation. Pharmacol. Rev..

[38] Tewari H.K., Gadia R., Kumar D., Venkatesh P. (2006). Sympathetic–Parasympathetic Activity and Reactivity in Central Serous Chorioretinopathy: A Case—Control Study. Investig. Ophthalmol. Vis. Sci..

[39] Karska-Basta I., Pociej-Marciak W., Chrząszcz M., Kubicka-Trząska A., Romanowska-Dixon B., Sanak M. (2020). Altered plasma cytokine levels in acute and chronic central serous chorioretinopathy. Acta Ophthalmol..

